# Sperm DNA Integrity Assessment: A New Tool in Diagnosis and Treatment of Fertility

**DOI:** 10.1155/2012/531042

**Published:** 2011-12-01

**Authors:** Mona Bungum

**Affiliations:** Reproductive Medicine Centre (RMC), Skane University Hospital, 205 02 Malmo, Sweden

## Abstract

Infertility affects 15% of all couples. Although male infertility factors with reduced semen quality are contributing to about half of all involuntary childlessness, the value of standard semen parameters in prediction of fertility *in vivo* and choice of proper method for assisted reproduction is limited. In the search for better markers of male fertility, during the last 10 years, assessment of sperm DNA integrity has emerged as a strong new biomarker of semen quality that may have the potential to discriminate between infertile and fertile men. Sperm DNA Fragmentation Index (DFI) as assessed by the flow cytometric Sperm Chromatin Structure Assay (SCSA) can be used for evaluation of sperm chromatin integrity. The biological background for abnormal DFI is not completely known, but clinical data show that DFI above 30% is associated with very low chance for achieving pregnancy in natural way or by insemination, but not *in vitro*. Already when the DFI is above 20%, the chance of natural pregnancy may be reduced, despite other sperm parameters being normal. Thus this method may explain a significant proportion of cases of unexplained infertility and can be beneficial in counselling involuntary childless couples need of *in vitro* fertilisation.

## 1. Introduction

In western countries up to one-forth of couples in reproductive age are seeking medical help for involuntary childlessness [1]. Despite the significant developments in the area of fertility seen during the last decades about one-third of these couples will be undiagnosed without any explanation to their problems.

Although, the traditional semen parameters concentration, motility, and morphology are a golden standard in diagnosing of male infertility it has become apparent that none of these parameters recommended by the Word Health Organization (WHO) [2] are sufficient for the prediction of male fertility capacity. As the WHO parameters only address few aspects of sperm quality and function the discriminative power in relation to fertility is quite low [3, 4].

As a result, there has for long been searched for better markers of male fertility.

During the last decades the use of assisted reproductive techniques (ARTs) has increased substantially [1, 5]. In particular intracytoplasmic sperm injection (ICSI) is used to an increasing degree. While in the beginning of the era of ICSI the indication for treatment was severe male factor infertility, now also couples with normal sperm quality request are treated with ICSI. However, IVF and ICSI are symptomatic treatments where only 25–30% of the treatments result in a delivery [6]. One explanation to this limited success can be the lack of markers to find the underlying causes to subfertility and also a lack of methods to identify the type of ART treatment providing the most optimal chances of pregnancy in a given couple.

During the last decade the search for better predictors of male fertility has resulted in an increased focus on the sperm DNA integrity [7, 8]. Now number of sperm chromatin integrity assays is available. Among the most frequently used are the Comet assay (single cell gel electrophoresis) [9], the TUNEL (terminal deoxynucleotidyl transferase-mediated dUDP nick end labelling) assay [10], the sperm chromatin dispersion (SCD) [11, 12], and the sperm chromatin structure assay (SCSA) [13, 14]. The clinical value of these different tests varies; however, SCSA, first described by Evenson et al. [13] is shown to be an independent marker of fertility *in vivo* and may also help in selection of the most effective ART treatment in each individual couple [15]. This paper will discuss how sperm DNA integrity assessment by help of SCSA can be used as a tool in diagnosis and treatment of infertility.

## 2. Aetiology and Diagnosis of Infertility

Involuntary childlessness, infertility can be a result of female, male as well as combined factors. It is a complex condition where often a mix of factors plays a role. In 20% of cases, the predominant cause is solely male related and, in another 30%, anomalies in both partners contribute to the childlessness [2]. Traditionally genital infections, endocrine disturbances, and immunological factors have been regarded as the most common causes of male subfertility. However, now more often genetic/molecular causes are identified as contributing factors [16], as,for instance, chromatin defects assessed as breaks in sperm DNA [7, 14, 17–19]. However, it is a fact that in 60–75% of the male-caused cases the aetiology of reduced semen quality remains unexplained and is therefore diagnosed as idiopathic infertility [2].

The golden standard in the diagnosis of male infertility or subfertility is an analysis of sperm concentration, motility, and morphology where WHO has set threshold levels for normality in regard to fertility [2]. However, this WHO sperm analysis is mainly performed by light microscopy of 100–200 spermatozoa and thus the analysis is biased because of a high level of subjectivity resulting in a high grade of intra- and interlaboratory variation [20, 21]. As a consequence a low predictive value of the WHO analysis is reported. Another drawback with the WHO parameters is that they only address few aspects of sperm quality and function. During the last decades, several other sperm function tests have been suggested to be used, including vital staining, hemizona assay, biochemical analysis of semen, antisperm antibody test, hypoosmotic swelling test, sperm penetration assay, reactive oxygen species (ROS) tests, and computer-assisted sperm analysis (CASA) [18]. However, as none of them have provided stable threshold values few are actually used in clinical routine [22].

Now an increasing amount of data demonstrate an association between sperm DNA damage and fertility [7, 14, 17, 19, 23–26]. It has been proposed that sperm DNA integrity could be a fertility predictor to be used as a supplement to the traditional sperm parameters [14, 15, 27].

## 3. Causes to Sperm DNA Damage

Spermatogenesis is a complex process [28] where damage of sperm chromatin structure can occur at any step (reviewed in [7]). DNA damage in sperm can be due to unrepaired DNA breaks during the spermatogenetic chromatin remodeling and packaging or abortive apoptosis during spermatogenesis. Among other suggested causes are the effect of endogenous endonucleases and caspases, exposure to a variety of genotoxic agents because of therapeutic reasons or because of occupational or environmental exposures, and finally, the action of oxidative sperm DNA damage [19]. Most likely often these factors are interrelating. Problems in the crossing-over process during spermatogenesis or deficiencies in the protamination process will likely make sperm more vulnerable to oxidative stress at a later occasion.

### 3.1. Remodeling and Packaging Problems

Meiotic crossing-over during spermatogenesis is associated with the programmed introduction of DNA double-strand breaks, expected to be ligated until the end of meiosis I [29]. Stage-specific introduction of transient DNA strand breaks during spermiogenesis has been described [30, 31]. DNA breaks are needed for transient relief of torsional stress, favouring the histone replacement with protamines during the final maturation form round to elongated spermatozoa [31, 32]. However, if and only if these physiological, normal temporary breaks are not repaired, DNA fragmentation in ejaculated spermatozoa or genetic mutations may occur [33].

### 3.2. Abortive Apoptosis

Another suggested aetiology of DNA damage is that breaks can arise through abortive apoptosis. Apoptosis of testicular germ cells occurs normally throughout life, controlling overproliferation [34]; however, it has been suggested an early apoptotic pathway, initiated in spermatogonia and spermatocytes, mediated by the Fas protein [35]. Sertoli cells in the testis express Fas ligand, which by binding to Fas leads to cell death through apoptosis [35].

### 3.3. Oxidative Stress

Oxidative stress (OS) caused by an imbalance between the antioxidant ability in seminal plasma and the production of reactive oxygen species (ROS) leading to the formation of oxidative products such as 8OHdG is the mechanism that probably most often lies behind sperm DNA defects. The sperm cell membrane is easily attacked by ROS with further detrimental effects on nuclear membranes as well as on sperm DNA [33]. Moreover, sperm lack antioxidants and DNA repair systems [36] and therefore protection of the offspring from the negative effects of male-induced DNA strand breaks is always dependant on the repair capacity of the oocyte and the early embryo. The main sources of ROS in semen are leukocytes and abnormal/dead spermatozoa in the semen [19, 37, 38]; however, increased scrotal temperature due to illness with fever [39–41], varicocele [42], increased age [43–47], and smoking [48–54] are also reported as sources.

## 4. Sperm DNA Integrity Assessment

Currently, four major tests of sperm DNA fragmentation are most frequently used, including the Comet assay (single cell gel electrophoresis) [9], the TUNEL (terminal deoxynucleotidyl transferase-mediated dUDP nick end labelling) assay [10], the sperm chromatin dispersion (SCD) test [11, 12], and the sperm chromatin structure assay (SCSA) [13, 14]. They all label single- or double-stranded DNA breaks; however unfortunately, most of the available techniques for detection of sperm DNA damage provide limited information on the nature of the DNA lesions detected and none of them enables us to depict the exact aetiology and pathogenesis of impairment of sperm DNA.

Comet assay is a fluorescence microscopic test, and TUNEL assay can be applied in both bright field/fluorescence microscopy and by flow cytometry. In Comet assay sperm cells are mixed with melted agarose and then placed on a glass slide. The cells are lysed and then subjected to horizontal electrophoresis. DNA is visualized with the help of a DNA-specific fluorescent dye and DNA damage is quantified by measuring the displacement between the genetic material of the nucleus comet head and the resulting tail. In the TUNEL assay, terminal deoxynucleotidyl transferase (TdT) incorporates labelled (by and large fluorescent) nucleotides to 3′-OH at single- and double-strand DNA breaks to create a signal, which increases with the number of DNA breaks. The fluorescence intensity of each analyzed sperm is determined as a “positive” or “negative” for sperm on a microscope slide. In a flow cytometer the fraction of positive sperm is represented by the cells above a threshold channel value on a relative fluorescent intensity scale.

SCSA is a flowcytometric test where sperm DNA breaks are evaluated indirectly through the DNA denaturability. The assay measures the susceptibility of sperm DNA to acid-induced DNA denaturation in situ, followed by staining with the fluorescence dye acridine orange [13, 14, 55]. By using a flow cytometer 5.000–10.000 sperm can be analyzed within few seconds and thus provide a less subjective result compared to the WHO analysis where only 1–300 cells are analyzed. Through a specific SCSA-software (SCSA-Soft) a scatter plot is created, showing the ratio of green and red sperm. The percentage of red sperm is called DNA fragmentation index (DFI) [14]. The sperm with the most intensive green colour is called high DNA stainable (HDS) sperm. It is still unclear precisely which mechanisms and types of DNA damage are lying behind DFI and HDS; however, it is believed that whilst DFI is related to the percentage of sperm with DNA breaks or protamine defects HDS is thought to represent immature spermatozoa [14].

So far, the SCSA is the only sperm DNA integrity assessment method which has demonstrated clear and clinically useful cut-off levels for calculating male fertility potential [13, 15, 27, 55]. The SCSA is a standardized test performed according to a strict protocol [14]. Apart from being subject to a very limited intralaboratory variation [56], however, the SCSA analysis has shown to be very robust to variation between laboratories. In an external quality control based on >180 samples, a high (*r* = 0.8) correlation was found between the values obtained by our laboratory and those from a control laboratory. Furthermore, the absolute DFI values obtained at two different laboratories, using different equipment, did not on average differ by >1% [57].

The advantage of SCSA is the objectivity of the test as well as the high reproducibility when running after the standardised protocol. Moreover, the clear cut-off levels in relation to fertility are maybe the most obvious benefit compared to other sperm DNA integrity tests. A disadvantage is that an expensive flow cytometer is required to run the analysis. Moreover, the test irreversibly damages spermatozoa; after analysis they cannot be used for fertilisation purposes.

Studies have demonstrated that these four sperm DNA integrity tests, the SCSA, TUNEL, Comet, and SCD assays, generally correlate moderately with each other (a coefficient of correlation between 0.4 and 0.7), which indicates that the tests likely are expressing different aspects of sperm DNA damage.

## 5. SCSA and the Chance of Pregnancy

Two population-based studies, including 165 and 215 couples respectively, [13, 55], have demonstrated that DFI as measured with SCSA is an excellent predictor of subfecundity in the normal population. In the interval of DFI 0–20%, the chance of spontaneous pregnancy was constant. When DFI was above 20% the chance of obtaining a spontaneous pregnancy was decreased and close to zero when the DFI level passed 30–40%. Even though DFI was below 20%, only 13% of all cycles resulted in a pregnancy. Therefore, in a normal population, not selected because of infertility problems, SCSA is a valuable tool to identify men who are at risk of not giving rise to a pregnancy. The same information is not possible to get from the traditional WHO sperm parameters. Even among men with low sperm concentration, poor motility or morphology there will be men with a certain potential of fertility [3].

In a case-control study of 137 infertile and 127 fertile men the risk of being infertile was found to be increased when DFI as measured by SCSA was above 20% in men with normal standard semen parameters, an odds ratio (OR) of 5.1, (CI: 1.2–23), compared to fertile controls [27]. If one of the WHO parameters was abnormal, the OR for infertility was increased already at DFI above 10% (OR 16, CI: 4.2–60). A DFI above 20% was found in 40% of the men with otherwise normal standard parameters. DFI was also shown to be an independent predictor of spontaneous pregnancy. Erenpreiss and coworkers found that 20% of the men with otherwise normal WHO sperm parameters had an SCSA-DFI above 20% [58].

The association between sperm DNA damage and the traditional semen parameters is shown to be only weak to moderate [57, 59]. It is also shown that 25–40% of infertile men may have normal standard sperm characteristics according to WHO criteria, but a DFI above 20–30% [15, 27, 58].

ART includes all technologies that involve the handling of sperm outside the body, as in intrauterine insemination (IUI), or handling of oocytes, sperm, and embryos as in *in vitro* fertilization (IVF) and ICSI [60]. In IVF, the spermatozoas ability to penetrate the zona pellucida of the oocyte is utilized. In ICSI, however, one single spermatozoon is selected and injected directly into the cytoplasm of the oocyte. Despite these obvious differences most studies report results from the three types of treatments together. In studies reporting the three treatment types separately, the number of patients included and thus the statistical power have been relatively low. The first SCSA study to indicate an association between sperm DNA damage and reduced pregnancy chances was published by Saleh et al. [61] who performed a small study where 12 of 19 couples had a DFI value as measured by SCSA above 28% and no pregnancy was obtained. Also, Boe-Hansen and coworkers in a study on 48 IUI couples found only two couples with a DFI value above 30%, and neither here pregnancy was obtained [62]. Recently, in a study of 387 IUI cycles, we have shown that even in IUI SCSA-DFI can be used as an independent predictor of fertility [15]. Whilst the proportion of children born per cycle was 19.0% when the DFI value was below 30%, those with a DFI value above 30% only had a take-home-baby rate of 1.5%. In this group the OR for delivery in relation to DFI <30% was 0.07 (95% confidence interval (CI): 0.01–0.48). In the same study, 388 IVF and 223 ICSI cycles were included but it was not possible to find any threshold value for DFI that could predict the result of the treatments. However, surprisingly when DFI exceeded 30%, the result of ICSI was significantly better than IVF (OR for delivery was 2.17 (CI 1.04–4.51)). These data are in agreement with other previous smaller reports using TUNEL or COMET assays, showing that sperm DNA damage is more predictive in IVF and much less so in ICSI [63, 64].

Although fertilization and embryo development may be independent of sperm DNA integrity, it has been suggested that the postfertilization development of the pre-embryo can be impaired by such incomplete or aberrant sperm DNA repair by the oocyte leading to early miscarriages (reviewed in [65]) or in the worst cases diseases in the offspring [8, 66, 67]. In our study of about 1000 couples no relationship was seen between sperm DNA fragmentation and unexplained pregnancy loss [15].

The other SCSA parameter, HDS, was neither in ours nor in other studies found to be of predictive value of pregnancy.

## 6. Clinical Recommendations

SCSA represents a valuable tool in diagnosis and treatment of infertility. The usefulness of the method is first of all relating to *in vivo *fertility (spontaneous pregnancy and IUI) and in particular in the many couples diagnosed with unexplained infertility. In 20% of men presenting with otherwise normal semen parameters, the SCSA-DFI is above 30% and in these couples the chance of pregnancy is close to zero [15] that is why they should be directly referred to IVF/ICSI. Through such a strategy change one-fifth of all couples normally referred to IUI can avoid the huge burden an unsuccessful ART treatment represents.

In as many as 40% of all unexplained infertile couples the explanation may be related to a high DFI. In a couple having a DFI between 20 and 30% time to pregnancy will be longer than in a couple with a DFI level below 20%. This is important information that should be utilized in counselling the couple. Combining SCSA-DFI with assessment of traditional WHO sperm parameters is shown to give a higher precision in the prediction of fertility. If one of the WHO sperm parameters is that abnormal fertility becomes reduced already when DFI exceeds the level of 10%, whereas the DFI level is above 30%, couple should be revised directly to IVF/ICSI. However, the status is that still very few clinics have implemented sperm chromatin integrity testing and therefore most couples seeking help for infertility problems are not aware of their actual potential sperm DNA defects. One reason is the necessity of expensive equipment as, for instance, a flow cytometer as well as time, cost, and expertise to perform the analysis. On the other hand, sperm samples can be shipped to and analyzed on a centralized SCSA laboratory.

Sperm DNA breaks are mainly thought to be a result of oxidative stress. Some reports demonstrating a positive effect of antioxidant therapy in men with a high DFI have been published; however, the study populations have been small and data conflicting [45, 68–73]. In the future SCSA may also have the potential to give indications for a causal treatment of disturbances of male fertility.

Development of new improved tests depicting the cause of sperm DNA damage should be the next step in using sperm DNA integrity testing as a tool in diagnosis and treatment of infertility.
